# Evaluation of Best Supportive Care and Systemic Chemotherapy as Treatment Stratified according to the retrospective Peritoneal Surface Disease Severity Score (PSDSS) for Peritoneal Carcinomatosis of Colorectal Origin

**DOI:** 10.1186/1471-2407-10-689

**Published:** 2010-12-22

**Authors:** Joerg OW Pelz, Terence C Chua, Jesus Esquivel, Alexander Stojadinovic, Joerg Doerfer, David L Morris, Uwe Maeder, Christoph-Thomas Germer, Alexander G Kerscher

**Affiliations:** 1 Department of General-, Visceral-, and Paediatric Surgery, University of Wuerzburg, Germany; 2 UNSW Department of Surgery, St George Hospital, Sydney, NSW, Australia; 3 Department of Surgical Oncology, St. Agnes Hospital, Baltimore, MD, USA; 4 Department of Surgery, Walter Reed Army Medical Center and the United States Military Cancer Institute, Washington, USA

## Abstract

**Background:**

We evaluate the long-term survival of patients with peritoneal carcinomatosis (PC) treated with systemic chemotherapy regimens, and the impact of the of the retrospective peritoneal disease severity score (PSDSS) on outcomes.

**Methods:**

One hundred sixty-seven consecutive patients treated with PC from colorectal cancer between years 1987-2006 were identified from a prospective institutional database. These patients either received no chemotherapy, 5-FU/Leucovorin or Oxaliplatin/Irinotecan-based chemotherapy. Stratification was made according to the retrospective PSDSS that classifies PC patients based on clinically relevant factors. Survival analysis was performed using the Kaplan-Meier method and comparison with the log-rank test.

**Results:**

Median survival was 5 months (95% CI, 3-7 months) for patients who had no chemotherapy, 11 months (95% CI, 6-9 months) for patients treated with 5 FU/LV, and 12 months (95% CI, 4-20 months) for patients treated with Oxaliplatin/Irinotecan-based chemotherapy. Survival differed between patients treated with chemotherapy compared to those patients who did not receive chemotherapy (p = 0.026). PSDSS staging was identified as an independent predictor for survival on multivariate analysis [RR 2.8 (95%CI 1.5-5.4); p < 0.001].

**Conclusion:**

A trend towards improved outcomes is demonstrated from treatment of patients with PC from colorectal cancer using modern systemic chemotherapy. The PSDSS appears to be a useful tool in patient selection and prognostication in PC of colorectal origin.

## Background

The majority of patients with peritoneal carcinomatosis (PC) from colorectal cancer present with unresectable disease at the time of diagnosis. The morbid nature and fatality peritoneal disease in patients with colorectal cancer is significant and the recent focus of clinical outcomes research. In a recent multi-centre prospective study of 370 patients with PC from non-gynecological malignancies, patients with colorectal cancer survived a median time of 5.2 months [1]. Research protocols using palliative systemic chemotherapy for PC have been conducted with encouraging tumor response rates, but overall survival remains poor [2,3]. The reported median survival after systemic 5-Fluorouracil/Leucovorin (5FU/L) based chemotherapy for PC of colorectal cancer can, under the best of circumstances, achieve median survival of only 5.2 to 12.6 months [4].

Modern systemic therapy regimens with combinations of cytotoxic and biological agents appear promising in clinical trials, demonstrating improved tumor response rates over older regimens ultimately translating into gains in both progression-free and overall survival in patients with metastatic colorectal cancer [5-10]. Nonetheless, the patient cohorts with Stage IV disease in these trials have failed to include patients with PC. The difficulties of including these patients are a result of the inability to image sub-centimetre peritoneal lesions and assess tumor response on the RECIST criteria. Hence, strictly speaking, this leaves this subgroup of patients with Stage IV colorectal cancer without any appreciable evidence of disease and the treatment response cannot be documented or monitored.

Aggressive surgical therapy has been shown to be promising when combined with hyperthermic intraperitoneal chemoperfusion (HIPEC). A multi-institutional registry study of 506 patients with PC of colorectal origin showed that median survival of up to 32 month can be attained with this aggressive multi-modality treatment approach in patients with limited peritoneal surface disease who are able to undergo complete cytoreduction [11]. More recently, Elias et al reported a 5-year survival rate of 51% and median survival of 63 months in patients with limited PC treated with oxaliplatin-based HIPEC [12].

The lack of specific data for patients with isolated PC represents a gap in the current literature. In the modern era of effective systemic chemotherapy, outcomes for this particular patient subset (limited PC of colorectal origin) need to be re-examined. Further, the considerable progress made in CS and HIPEC in peritoneal carcinomatosis has not rightfully translated into routine clinical practice. Debate over the appropriateness of CS and HIPEC as a treatment strategy without concrete and replicable data from randomized trials, together with concerns over aggregate treatment-related morbidity and mortality ranging from 14% to 55% and 0% to 19%, respectively [4], have hampered the ability to reach a treatment consensus amongst the general oncology community. To evaluate the effectiveness of systemic chemotherapy, we report the results of a single institution experience of systemic chemotherapy for PC from colorectal cancer with stratification according to the peritoneal surface disease severity score (PSDSS) to elucidate stage-specific outcomes that may guide clinical treatment decision for patient-specific delivery of therapy.

## Methods

### Cohort Definition

Between January 1 1987 and December 31 2006, patients with colorectal cancer treated at the University of Wuerzburg Medical Centre were identified from the Wuerzburg Institutional Database (WID). In our institution, the surgical peritoneal surface malignancies program (including debulking surgery and HIPEC) was initiated in September 2008. Patients were included if they had intraoperatively confirmed peritoneal carcinomatosis either at the time of initial presentation or at time of recurrence with histological diagnosis of tumor from colorectal origin. The exclusion criteria were for peritoneal carcinomatosis from non-colorectal origin, patients died within 30 days after exploration or having more than three extra abdominal metastases.

### Data Source

The WID is a central data repository that is expanded prospectively on a daily basis with clinical, operative, and research data of patients who were evaluated and treated at the University of Wuerzburg Medical Centre. Data available within the WID include patient demographics, histological diagnoses that are based on International Classification of Diseases coding standards, physician and hospital billing data, inpatient admission and outpatient registration data, operating room procedures, laboratory results, and computerized pharmacy records. The WID undergoes continuous cross platform integration with the Comprehensive Cancer Registry to ensure updated follow-up information for identification of deceased patients. Inpatient and outpatient records of all identified patients were reviewed retrospectively to extract information regarding type and duration of chemotherapy, sites of metastatic disease at presentation and disease status at last follow-up.

### Retrospective Peritoneal Surface Disease Severity Score (PSDSS)

The retrospective PSDSS was estimated based on the three most important prognostic indicators; clinical symptoms, extent of carcinomatosis based on the tumor burden (analog PCI) and tumor histopathology [13]. Each of these three categories was classified into three sub-categories based on the severity of each clinicopathological factor:

1. Clinical Symptoms; none, mild (weight loss < 10% of body weight, mild abdominal pain, asymptomatic ascites) or severe (weight loss ≥ 10% of body weight, unremitting pain, bowel obstruction, symptomatic ascites).

2. Extent of Carcinomatosis intraoperatively; limited (analog PCI < 10), moderate (analog PCI 10 to 20) or extensive (analog PCI > 20).

3. Tumor histopathology of the primary tumor; well to moderately differentiated without positive lymph node, moderately differentiated with positive lymph nodes or poorly differentiated and/or signet ring (Table 1).

**Table 1:** Estimation of Peritoneal Surface Disease Severity of Patients with Colorectal Cancer Peritoneal Carcinomatosis

Peritoneal Surface Disease Severity Score for Colorectal Cancer		
**Clinical Symptoms**	**Extent of Carcinomatosis**	**Primary Tumor Histopathology**

No Symptoms	PCI < 10	Well or Moderately Differentiated and N0
**0 points**	**1 Point**	**1 Point**

Mild Symptoms	PCI 10 to 20	Moderately Differentiated and N1 or N2
**1 Point**	**3 Points**	**3 Points**

Severe Symptoms	PCI > 20	Poorly Differentiated or Signet Ring
**6 Points**	**7 Points**	**9 Points**

The impact of these clinicopathological variables derived from the patient's clinical presentation at the time of evaluation for treatment, radiological assessment of the extent of carcinomatosis, and the tumor histopathology. This was scored as stages I to IV based on the summation of the arbitrary scores for each of the three clinicopathological staging parameters based on our clinical experience: PSDSS Stage I < 4; PSDSS Stage II = 4-7; PSDSS Stage III = 8-10; PSDSS Stage IV > 10.

### Follow-Up and Outcomes

Treatment was grouped according to the type of systemic chemotherapy regimen; no chemotherapy (best supportive care), 5-Fluorouracil/Leucovorin (5FU/L), or modern chemotherapy (Oxaliplatin/Irinotecan-based) with or without biological agents (Bevacizumab/Cetuximab/Panitumumab). All patients were followed every 3 months. Helical contrast enhanced computed tomography (CT) was performed every 6 months. Follow-up data was obtained from the referring physicians, phone calls and/or emails from the patients, or the cancer registry. All deaths in this study were disease-related, attributable to progressive colorectal cancer. The primary study endpoint was from the time of diagnosis of peritoneal carcinomatosis to the time of death (overall survival). Follow-up data recorded included the data of the status of the patient (alive with disease, alive without disease and dead of disease).

### Statistics

The data collected were analyzed using JMP software (JMP^® ^, Cary, NC Version 7) software. The patient characteristics were reported using frequency and descriptive analyses. The Kaplan-Meier method was used to analyze survival. Univariate analysis (log-rank) was performed to determine the clinicopathological factors affecting survival, including the PSDSS stage. All factors correlating with outcome having p < 0.10 on univariate were entered into a Cox proportional hazards regression model for multivariate analysis. The median time to death was defined as the time where 50% of patients have died. P < 0.05 was considered statistically significant.

## Results

### Patient Characteristics

One thousand nine hundred and twenty patients with colorectal cancer underwent a laparotomy during the study period. Peritoneal carcinomatosis was observed in 240 patients (13%); 98 patients (42%) at initial diagnosis and 142 patients (58%) at time of recurrence. Ten patients (2%) died from surgical complications during the immediate post operative period, eight patients (3%) died prematurely of non-cancer related reasons, 20 patients (8%) had incomplete records in the database, and 35 patients (15%) with more than 3 extra abdominal metastasis were excluded from study. In total, 167 patients formed the cohort of this study.

The median age was 63 (range, 22 to 88) years. Sixty-four patients (38%) had isolated peritoneal carcinomatosis. Aside from peritoneal carcinomatosis, other sites of metastasis include the liver or lung in 67 patients (40%) and 36 patients (22%) had peritoneal carcinomatosis with bone or brain metastasis. The detailed patient characteristics are presented in Table 2.

**Table 2:** Characteristics of patients with peritoneal surface malignancy of colonic origin (n = 167)

Characteristics	N	% of Total
Male	93	56

Age < 50 Years	74	44

Mean Age (Years ± SD)	63 ± 17	

Disease Presentation		
Synchronous	52	33
Metachronous	115	67

Location		
Colon	123	73
Rectum	44	27

Site of Metastases		
Peritoneum Only	64	38
Peritoneum+Lung/Liver	67	40
Peritoneum+Other	36	22

Systemic Chemotherapy		
None	83	50
5FU/L	42	25
Modern	42	25

PSDSS Stage		
Stage I	6	4
Stage II	53	31
Stage III	33	20
Stage IV	75	45

### Survival Analysis

**Figure 1:**
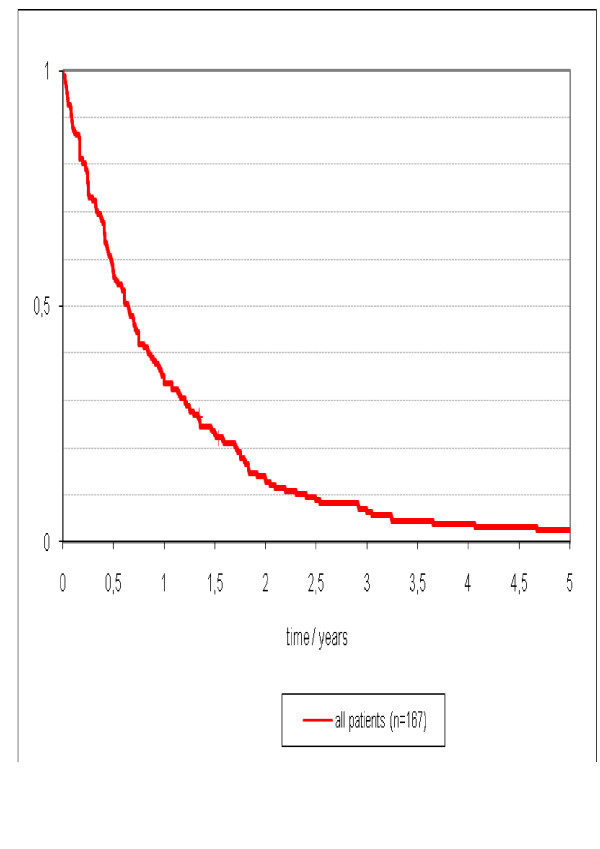
**Survival of 167 patients with isolated peritoneal carcinomatosis or peritoneal carcinomatosis as a combined site of disease with less than two other metastatic sites**.

The median follow-up time from diagnosis of peritoneal carcinomatosis to last clinical follow up was 8 (range, 1 to 112) months. At the time of analysis, 163 patients (98%) have died of disease and there were four survivors (2%) who are alive without disease. The median follow-up in these four survivors was 78 (range, 43 to 112) months. The overall median survival was 8 (95%CI 6 to 9) months and the 3- and 5-year overall survival was 6% and 3% respectively (Figure 1).

### Impact of Chemotherapy Treatment on Outcomes

Eighty-three patients (50%) had no chemotherapy treatment and received best supportive care only. Forty-two patients (25%) received 5FU/L chemotherapy and forty-two patients (25%) received modern chemotherapy of which eight patients (5%) had biological agents in combination with modern chemotherapy. The median duration of chemotherapy treatment was 18 (range, 0 to 115) weeks.

**Figure 2:**
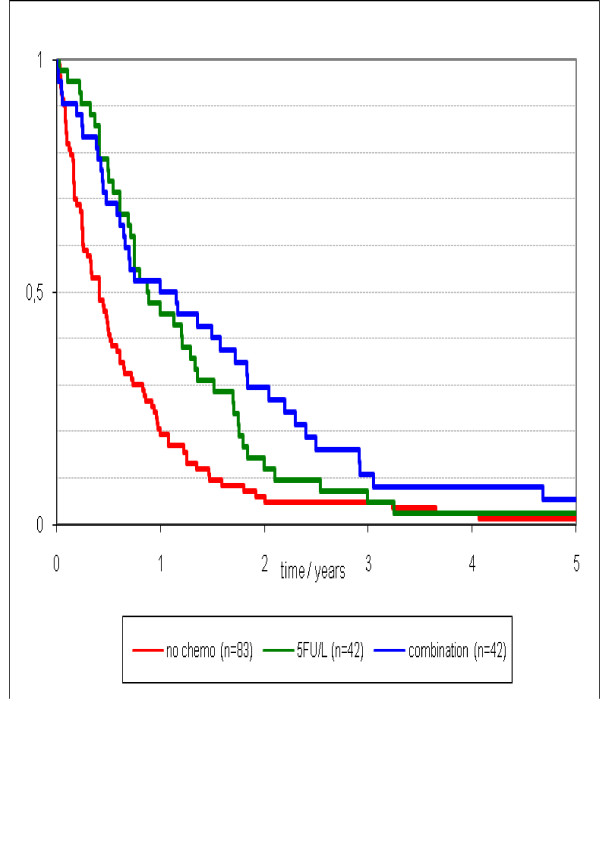
**Survival stratified by type of chemotherapy treatment (no chemo vs. 5FU/L and modern systemic chemotherapy; p = 0.026)**.

The median survival was 5 (95%CI 3 to 7) months in patients receiving best supportive care, 11 (95%CI 6 to 15) months for patients treated with 5 FU/L, and 12 (95%CI 4 to 20) months for patients treated with modern chemotherapy. The median survival differed significantly in patients who received chemotherapy versus those who received best supportive care (p = 0.026), however, outcomes did not differ between patients treated with 5FU/L or modern chemotherapy (p > 0.05) (Figure 2).

### Stratifications According to the retrospective PSDSS

Six patients (4%) were scored as PSDSS Stage I, 53 patients (32%) as PSDSS Stage II, 33 patients (20%) as PSDSS Stage III and 75 patients (45%) as PSDSS Stage IV. The detailed treatment type in patients classified according to the PSDSS is shown in Table 3. Treatment differed between the four PSDSS Stages (p = 0.02).

**Table 3:** Analysis of chemotherapy treatment by PSDSS staging

Chemotherapy Treatment	PSDSS Stage I (n = 6)	PSDSS Stage II (n = 53)	PSDSS Stage III (n = 33)	PSDSS Stage IV (n = 75)
Best Supportive Care	4	15	12	52

5FU/L	1	19	10	12

Modern Systemic Chemotherapy	1	19	11	11

**Figure 3:**
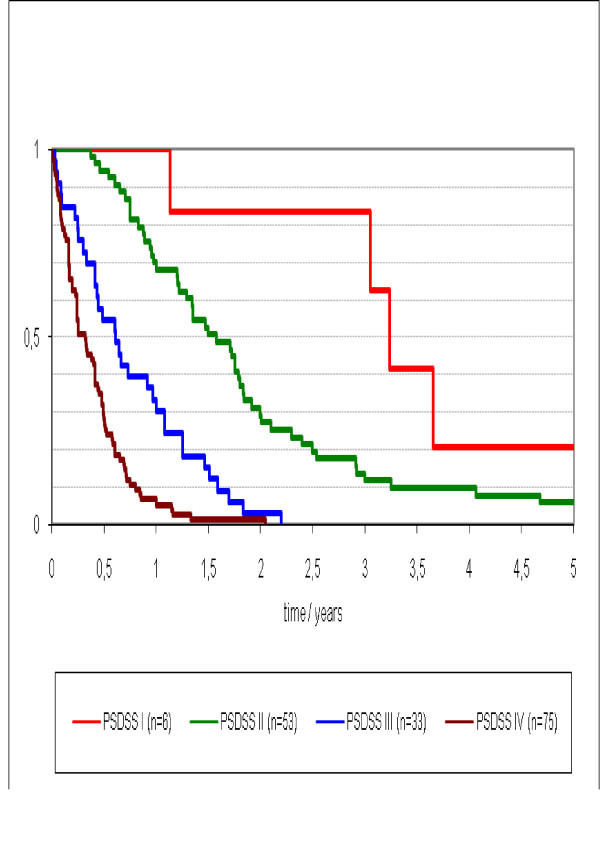
**Survival according to Peritoneal Surface Disease Severity Score Stage I to IV**.

**Figure 4:**
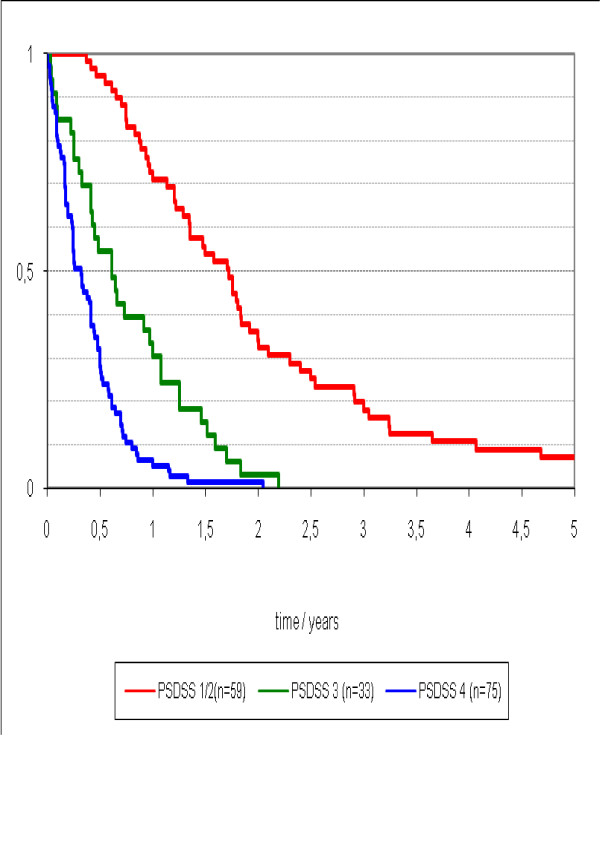
**Survival stratified by PSDSS Stage I/II, PSDSS Stage III and PSDSS Stage IV**.

Median survival differed stage-wise was 4 (95%CI 2.7 - 5.1) months for PSDSS Stage IV, 7 (95%CI 4.4 - 10.3) months for PSDSS Stage III, 19 (95%CI 13.8 - 24.1) months for PSDSS Stage II, and 39 (95%CI 34.2 - 42.4) months for PSDSS Stage I (p = 0.003) (Figure 3). The median survival of all patients with PSDSS Stage I/II was 22 (95%CI 14.2 - 26.7) months and for PSDSS Stage III/IV was 5 (95%CI 4.2 - 7.2) (p < 0.001) (Figure 4).

**Figure 5:**
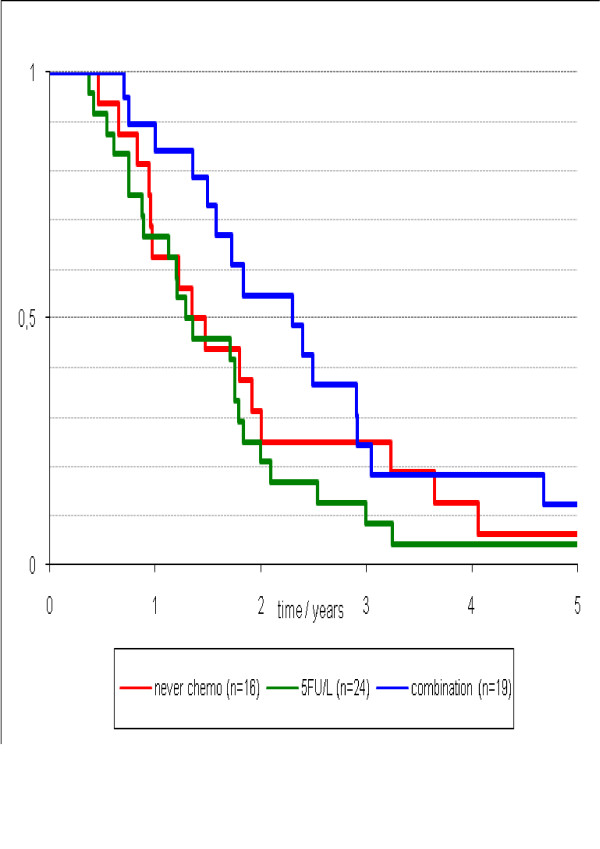
**Survival stratified by PSDSS Stage I/II by no chemotherapy, 5FU/L, modern combination systemic chemotherapy (no chemo v.s. 5FU/L and combination**.

In the PSDSS Stage I/II patients (n = 59) who received best supportive care, the median survival was 16 (95%CI 12.8 - 24.0) months; for those who received 5FU/L, the median survival was 16 (95%CI 13.7 - 22.8) months, and for patients treated with modern systemic chemotherapy, the median survival was 28 (95%CI 17.1 - 38.2) months (p = 0.12) (Figure 5). For a subgroup of patients with isolated PC with PSDSS Stage I/II (n = 20), the median survival was 21 (95%CI 16.6 - 24.8) and not different compared to the whole group.

Analysis of overall survival from diagnosis of carcinomatosis to last follow-up in uni- and multivariate analyze is shown in Table 4

**Table 4:** Analysis of overall survival from diagnosis of carcinomatosis to last follow-up in 167 patients with peritoneal carcinomatosis of colonic origin

Characteristics	n	Median Survival (Months)	P (Univariate)	P (Multivariate) RR [CI]
**Sex**			0.45	
Male	93	8		
Female	74	8		

**Age (Years)**			0.58	
< 60	85	8		
≥ 60	82	8		

**Disease Presentation**			0.45	
Synchronous	52	7		
Metachronous	115	8		

**Location**			0.07	
Colon	123	8		
Rectum	44	7		

**Site of Metastases**			0.15	
Peritoneum Only	64	7		
Peritoneum+Lung/Liver	67	8		
Peritoneum+Other	36	9		

**Systemic Chemotherapy**			0.003	
None	83	5		
5FU/L	42	11		
Modern	42	12		

**Clinical Symptoms**			< 0.001	
Asymptomatic	55	18		
Mild	78	6		
Severe	34	3		

**Extent of Carcinomatosis**			0.002	
PCI < 10	103	6		
PCI 10 to 20	47	11		
PCI > 20	17	4		

**Histopathology**			0.003	
Well Differentiated	19	7		
Moderately Differentiated	89	11		
Poorly Differentiated/Signet	59	6		

**PSDSS Stage**			0.003	0.001
Stage I	6	39		2.1 [1.3 - 4.9]
Stage II	53	19		
Stage III	33	7		
Stage IV	75	4		

**PSDSS Stage**			< 0.001	< 0.001
Stage I/II	59	22		2.8 [1.5 - 5.4]
Stage III/IV	108	5		

## Discussion

Cytoreductive surgery (CS) combined with intraoperative hyperthermic intraperitoneal chemotherapy (HIPEC) is a treatment option for selected patients with peritoneal carcinomatosis (PC) from colorectal cancer. There has been enormous interest in the literature about this multi-modality therapeutic approach for a disease that has been associated with poor outcome. Phase II studies have demonstrated that CS combined with HIPEC is associated with an improved survival in patients with limited PC amenable to complete cytoreduction when compared to historical controls which were treated palliatively with systemic chemotherapy alone [14]. In 2004, a multi-institutional registry from 28 international treatment centres demonstrated that the median survival was 19 months and 3-year survival was 39% in 506 patients with CRPC who were treated with CS and HIPEC. These early outcomes are encouraging; however, treatment-related morbidity and mortality contribute to continued concern over the feasibility of this aggressive multi-modality therapy approach [11]. With continued specialty-centre experience, the patient selection process has improved. A recently published consensus statement emphasized the critical importance of proper patient selection to identify only suitable candidates for treatment to ensure that appropriately selected candidates receive and benefit from treatment, and unsuitable candidates are not subjected to the morbidity of a procedure unlikely to improve patient outcome [15].

By redefining and optimizing the patient selection process, treatment of patients with only limited PC has been shown to provide potentially curative oncological treatment. Elias et al. reported in a comparative trial a median survival of 62.7 months for patients with limited PC treated with CS and HIPEC compared to a median survival of 23.9 months in patients treated with palliative surgery and systemic chemotherapy alone [12]. Although, the survival results in this study reflect a highly selected group of patients, the impressive survival results support the concept that CS/HIPEC is a potentially curative treatment strategy and if performed in patients with limited PC, cure can be attained with high likelihood. If the extent of PC is not controlled through complete cytoreduction, CS and HIPEC may still prove beneficial; however, its role in the current era of modern systemic chemotherapy may require further investigation.

As part of the efforts to identify patients with PC that are suitable candidates for CS/HIPEC, Pelz et al proposed and validated a scoring system (Peritoneal Surface Disease Severity Score) that stages patients with PC taking into consideration the clinicopathological markers that predict for treatment outcome [13]. In an analysis of patients who underwent a complete cytoreduction, patients who were staged as PSDSS Stage I and Stage II were shown to have a 3-year overall survival of 60% to 80%. Although the study was limited by the follow-up time, the early results were promising and the long-term outlook depicted in the Kaplan-Meier curve showed a trend towards long-term survival [16].

In the present study, we used a retrospective PSDSS, because the PCI, described by Sugarbaker, was published first in 1995. The retrospective evaluation of the PCI is very difficult. For this reason, we used the term low, moderate and extensive to describe the tumor burden, analog to the PCI <10, 11-20 and > 20.

The findings of the current study affirm the premise that peritoneal carcinomatosis is a foremost cause of disease-specific mortality in patients with metastatic colorectal cancer. Patients with isolated PC, PC with liver/lung metastasis, or PC with brain/bone metastasis, predictably experienced early demise (p = 0.15), with an overall median survival of 5.0 months. The poor survival results reflect a subgroup of patients observed routinely in clinical practice for whom treatment options are limited. The biologically aggressive nature of PC impairs the functional status of patients to an extent that makes them eligible only for palliative, best supportive care only. It also remains unfortunate that, although modern systemic chemotherapy have improved survival in patients with metastatic colorectal cancer, the analysis in our study did not show a difference in outcomes between treatment with 5FU/L compared to modern chemotherapy in patients with PC (Figure 2). However, the authors do acknowledge that the number of patients receiving modern systemic chemotherapy, especially in combination with biological agents, in the current study are small, and further studies involving a larger cohort of patients is required to elucidate the true treatment effects.

By demonstrating a stage-wise difference in survival stratified according to the PSDSS, it appears that this staging system is of clinically meaningful prognostic utility in patients with peritoneal carcinomatosis. It is important to emphasize the marked contrast in survival outcomes between patients with PSDSS stage I/II and stage III/IV PC. Further, in patients with isolated PC who are PSDSS stage I/II, the median survival was 21 months. This survival result is comparable to current survival data from randomized trials of metastatic colorectal cancer that encompasses the use of modern systemic chemotherapy in combination with biological agents [17-19]. To draw upon the favourable prognosis of this group of patients, it is likely that patients with no symptomatology, low volume peritoneal disease, and favourable tumor biology, may derive the maximal benefits of the effective CS/HIPEC treatment strategy.

## Conclusions

In conclusion, our data demonstrates that peritoneal carcinomatosis remains a fatal condition in patients with metastatic colorectal cancer and it appears to be the dominant determinant of outcome. Treatment with systemic chemotherapy, especially modern agents is likely to be beneficial in patients with PC of colorectal origin. The optimal treatment results based on current evidence may be attained through careful selection of patients with a "favourable prognosis" for multi-modality therapy in whom the benefits of treatment outweigh the associated risks, for example, patients with PSDSS stage I/II, to undergo radical surgical cytoreduction in combination with hyperthermic intraperitoneal chemotherapy in an effort to obtain potentially curative disease clearance and extend the overall survival.

## Competing interests

Dr. Terence C. Chua is a surgical oncology research scholar funded by the St George Medical Research Foundation. The other authors indicated no potential conflicts of interest.

## Authors' contributions

JOWP, TCC, JE, AS, DLM and AGK have developed the study concept. UM was responsible for statistical considerations. JOWP, JD, CTG and AGK followed the patients and collected the data. JOWP, TCC and AGK drafted the manuscript. All authors contributed to and approved the final manuscript.

## Pre-publication history

The pre-publication history for this paper can be accessed here:

http://www.biomedcentral.com/1471-2407/10/689/prepub
